# Knowledge and compliance with standard precautions among registered nurses: A cross-sectional study

**DOI:** 10.1016/j.amsu.2021.01.058

**Published:** 2021-01-29

**Authors:** Ibrahim Al-Faouri, Suhib Hussein Okour, Nemeh Ahmad Alakour, Nasr Alrabadi

**Affiliations:** a Department of Community and Mental Health Nursing, Faculty of Nursing, Jordan University of Science and Technology, Irbid, 22110, Jordan; b Department of Maternal and Child Health Nursing, Faculty of Nursing, Jordan University of Science and Technology, Irbid, 22110, Jordan; c Department of Pharmacology, Faculty of Medicine, Jordan University of Science and Technology, Irbid, 22110, Jordan

**Keywords:** Compliance, Infection control, Jordan, Knowledge, Nurses, Standard precaution guidelines

## Abstract

**Background:**

Nurses and patients are often exposed to various types of infections during their clinical practice. Knowledge and compliance with standard precautions are essential to prevent hospitals associated infections and protect patients as well as medical workers from exposure to infectious agents.

**Aims:**

This study aimed to assess the level of knowledge, level of compliance, and associated factors toward compliance with standard precautions among registered nurses in the North of Jordan.

**Methods:**

A cross-sectional study was conducted at three hospitals in the North of Jordan. Two were public hospitals and one was a university-affiliated hospital. A questionnaire concerning the knowledge and compliance with the standard precaution guidelines was distributed among 300 registered nurses of whom 266 completed the questionnaire (response rate 88.7%).

**Results:**

53% of the participants were from governmental hospitals and 57.1% were females. The age median of them was 30 years (IQR = 28–32). The majority of the participants were medical/surgical RNs (33.1%) while only 8.3% of them were from the pediatric/gynecology departments. The overall knowledge score was 16.27 (SD = 3.15), and the total compliance score was 49.15 (SD = 12.36). Besides, the study showed a moderate positive correlation between the level of knowledge, experience in years, and the standard precautions compliance (r = 0.387, p = 0.01), (r = 0.341, p = 0.01), respectively.

**Conclusions:**

standard precautions are the basic level of the infection control process. The participants may possess satisfactory knowledge and compliance levels. However, more training programs and more focusing on the standard precautions by educational institutes are needed for nurses to improve their knowledge and compliance with infection-control standard precautions.

## Highlights

• Knowledge and compliance with standard precaution guidelines are essential during the era of the COVID-19 pandemic.
• Nurses from Jordan may possess satisfactory knowledge and compliance levels.
• More training programs and more focusing on the standard precautions by nurses' educational institutes is needed.

## 1 Introduction

Health-care associated infections (HAIs) considered one of the serious problems that face healthcare providers while handling patients' services. Those infections are common causes of morbidity and mortality among hospitalized patients [1]. Improving patient safety has received too much attention worldwide and one of the first goals of the World Health Organization's World Alliance for Patient Safety is to reduce HAIs [[2], [3], [4], [5], [6]]. According to the Center for Disease Control and Prevention in 2011, standard precautions were defined as “the minimum infection prevention measures that should be applied to all patient care” regardless of their suspicion or confirmation of infection status of the patients, which are used in any setting where health care is delivered [7]. These precautions should be applied at any setting where health-care services are delivered and always assuming that patients' blood, body fluid, secretions, and excrements have infectivity potentials [8,9]. When nurses providing nursing care for patients, they are exposed to the patient's body fluids, blood, and they may use needles that might be contaminated with several types of infectious pathogens. This may increase the risk of acquiring infections. Consequently, knowledge and compliance with standard precautions among nurses are important to reduce the incidence of those secondary infections [10,11]. However, the reality of adopting standard precautions in clinical settings is far away from what is recommended and has been proved to be somewhat problematic [12,13]. In fact, despite the awareness of the importance of standard precautions in reducing the transmission of infectious agents in the workplace, low compliance rates among health care personnel have been reported worldwide [12,14,15].

The factors affecting compliance with standard precautions may be related to environmental factors like materials, equipment availability, or maybe related to manager lack of commitment or individual factors like knowledge and experience [[16], [17], [18], [19]]. Therefore, to increase compliance with standard precautions and to eliminate the factors which have a negative influence on compliance, there should be critical behavioral changes in nursing practice. Those behavioral changes can involve the combination of education, motivation, and organizational changes. Besides, promoting compliance with standard precautions should mainly involve behavioral, environmental, and management actions, and should be going beyond the individual focus, which most institutions adopt by blaming the victim [[20], [21], [22], [23]]. It is very important to take into consideration that the incidence of infectious blood diseases and the spread of non-blood infectious diseases, such as those transmitted across the respiratory system, has increased [6,12]. Concomitantly, there is great emphasis on following the standard precautions among all medical workers. Following the standard precaution guidelines, which are easy and simple to follow, can reduce the transmission of many types of infectious diseases. As well, it can reduce the hospitalization period and the economic burden for treating these diseases. In England, the annual financial cost topped 1.3 billion Euros in 2008. While in the USA, about 3.5 billion Euros and 7 billion Euros in Europe for treating HAIs among patients [24].

Few studies were conducted in Jordan concerning the knowledge and compliance with standard precautions among nurses [25,26]. Therefore, this study aimed to investigate the level of knowledge and compliance with standard precaution guidelines among Jordanian registered nurses, identify the relationship between the level of knowledge and the level of compliance, and to identify the factors that affect the compliance with those guidelines. Consequently, identifying the avenues for improvements in Jordanian nursing practice.

## 2 Methodology and study design

A cross-sectional design was used to assess the level of knowledge and compliance with standard precautions guidelines among registered nurses in northern Jordanian hospitals. The data was collected from three major hospitals between the 15th of April and the 15^th^ of May 2020. The first one is King Abdullah University Hospital (KAUH), which is a teaching hospital affiliated with Jordan University of Science and Technology. The second Hospital is “Prince Basma Hospital”, a governmental hospital that is considered the largest public hospital in the north of Jordan. To cover the northern area of Jordan, participants from “Al-Mafraq public-Hospital” were also recruited in this study. It is a public hospital that serves a large area in the northeast of Jordan. The participants of this study were Jordanian Registered Nurses who were employed at the selected hospitals and completed at least a Bachelor's degree in nursing (4 years) or more, and who have experience at least one year of clinical practice and fully cooperative to complete the survey. According to Cohen's table for statistical analysis and sample size (Cohen, 1992), the sample size was determined based on a power of 0.80 and a level of significance (P = 0.05) at a moderate effect size. So, the minimum sample size is 255 participants. To avoid attrition and dropout from the study, the sample size increased to 300 participants [27].

The convenience sampling technique was used to collect data from the targeted population. This type of sampling technique is considered suitable for this study because of the easiness of selecting participants and the availability or easy access to participants. The data was collected over any duty shifts to increase the representativeness of the sample. Institutional Review Boards (IRB) approval was obtained from the Jordan University of Science and Technology (J.U.S.T) before starting data collection for this study. Participants were given a consent form with the questionnaire. Besides, participation was anonymous and participants were informed that participation in the study is voluntary and they can withdraw from the study at any time during data collection without penalties. Also, the selected hospitals were asked for their approval to conduct the study in their settings. The directors of nurses and head nurses in each hospital were met and the aim of the study was explained to them. After that, all eligible participants were invited to participate in this study, while the consent form, study aim, data collection methods, and instruments used were explained to all participants.

The questionnaire consisted of three main domains in addition to the socio-demographical variables. The first domain included eight questions about standard precaution practice. These questions were about the last injury by polluted instruments or exposure to blood or body fluids in the last six months. The frequencies of reporting injuries when they happened and the reasons for not reporting them were recorded. Also, there were two questions about if nurses received standard precautions training and the desire to have this training. The last two questions were about the sharp disposal box and vaccination status against the Hepatitis B virus. The second domain was about standard precautions knowledge. This part was first developed by Askarian et al. [16,17] and modified by Yang Lou et al. [8]. This scale consists of 20 items about basic concepts and activities related to standard precautions knowledge. The responses for this scale were (yes) or (no) or (uncertain). The “Yes” answer was given 1 point and “no” or “uncertain” were given 0 points. The total score was 20 and the higher the score was an indicator for increasing the level of knowledge regarding standard precautions guidelines. The third domain was about participants’ compliance with standard precautions guidelines. This part has been developed previously [8,16]. According to this scale, there were 20 items with a 5-points Likert scale from 0 to 4, where 0 = never, 1 = seldom, 2 = sometimes, 3 = usually, and 4 = always. The total score ranged from 0 to 80, in which a higher score indicated higher compliance with standard precautions. Finally, this cross-sectional study has been reported in line with the STROCSS Criteria [28]. As well, this study was registered with a Research Registry and the unique identifying number is researchregistry6454 [29].

### 2.1 Statistical analysis

The collected data were analyzed using SPSS version 22 for windows. Descriptive statistical analysis was applied for participant's sociodemographic data, level of knowledge, and level of compliance. Spearman rho correlation was used to test the relationship between the level of knowledge and the level of compliance to standard precautions guidelines. For the comparison between nurses' level of knowledge and level of compliance, and the socio-demographical variables, the Kruskal Wallis test, and Mann- Whitney *U* test were used. Standard multiple regression was performed to add more understanding about the relationships between the compliance and other predicting variables. A significant *p*-value was set at (P ≤ 0.05).

## 3 Results

### 3.1 Participants' characteristics

Out of the 300 participants who initially joined the study, 266 of them completed both the knowledge and compliance sections of the standard precautions questionnaire with a response rate of 88.7%. Participants' ages were almost consistent and most of the participants were females (57.1%). Most of the study participants (90.6%) hold a Bachelor's degree in nursing and the rest of the participants (9.4%) hold a Master's degree. Regarding the professional ranks, almost equal numbers of participants were registered nurses or in-charge/senior nurses (48.5% and 47.0%, respectively). However, a few of them (4.4%) were head nurses/nurse managers. Participants were also distributed over different working units. The majority were working in the medical/surgical unit (33.1%), the operation unit (9.3%), the emergency unit (25.1%), and the intensive care unit (24.1%) while only 8.3% of them worked in the pediatric, gynecology, and obstetric units. It was noted that the participants in the KAUH have significantly (p < 0.001) longer clinical experience than participants from other hospitals.

### 3.2 Knowledge of standard precautions guidelines

Most participants' responses tended towards accepting all practices except the last two items which focused on safety precaution for patients with tuberculosis, Varicella, intestinal infection, and skin infection, which were recorded as rejection/uncertain responses by participants. Regarding the total knowledge score of standard precaution where higher scores indicate a better understanding of the concept, participants showed a mean total knowledge score of 16.27 out of 20 with a standard deviation SD = 3.15. This result indicated a high level of knowledge, which was also found compatible with the responses in the individual questions. It was also noted that the internal consistency of participants' responses using Cronbach's Alpha test was also acceptable (0.79). Looking at scale sub-items in the knowledge questionnaire, all these items were positively worded. We found that 95.1% of participants knew what standard precautions and a total of 94.0% agreed that standard precautions applied to all patients. Washing hands after contacting blood, body fluids was 91.4%, and if contacting different patients was 92.1%. Moreover, 87.2% wore gloves in any procedure that might induce blood or body fluids, and 88.7% of the total participants reported changing their gloves if contacting different patients. 89.1% of the nurses were knowing that they should wear a face mask when dealing with a splash of blood or body fluids, however, a lesser number of them showed the same response regarding goggles use (75.6%). More than half of the participants did not know or were uncertain about caring for patients with tuberculosis or skin infection disease which may need extra precautions such as airborne or contact precautions.

### 3.3 Compliance with standard precautions guidelines

The results of compliance with standard precautions were provided. Most of the responses were above the midpoint (sometimes-always) except 3 questions (15, 16, and 17) which focused on wearing the protective eye patch, suit, and cap or shoe shade in the operation and their relation to spreading blood, body fluids, or body excretions.

Regarding the compliance total score, it was found that all nurses scored a mean of 49.15 out of 80 in their compliance with standard precautions. This result indicated an intermediate level of compliance because it falls around the midpoint of the total score which is 40. The reliability test revealed a high level of internal consistency at 0.93 using Cronbach's alpha statistic. Only 18.4% of participants always wash their hands when contacting different patients, and 19.2% if taking off gloves, and 23.3% washing hands immediately if contacting any blood, body fluids, and secretions. Regarding nursing compliance to wearing gloves when performing procedures, it was found that better compliance was when contact impaired skin and when changes dressing 25.2%. While the lowest compliance with wearing gloves was found when giving intramuscular injections (4.9%) followed by venous puncture (3.4%). The result also showed that 13.2% of the participants wearing the mask, and only 6% wearing goggles in the procedures that might include blood or body fluids. For needles recapping compliance, it was found that only 9.8% were fully compliant and won't recap the used needles, while 35.3% of them reported that sometimes they recapped the used needles.

### 3.4 Comparisons of nurses' knowledge according to sociodemographic variables

As shown in Table 1, participants from the KAUH scored higher in knowledge score (18.7/20) compared to registered nurses from both Prince Basma and Al Mafraq hospitals (13.8, 14.4/20, respectively) with (p < 0.001). On the other hand, there was no significant difference between male and female participants concerning the total knowledge score. Likewise, there was no significant difference between nurses in different professional ranks regarding the knowledge scores. However, nurses who were holding the Master's degree scored significantly higher in knowledge score (18.12/20) compared to nurses who were holding the Bachelor's degree (16.08) (P = 0.002). This result may not be generalized due to the small number of participated nurses who were holding the Master's degree. Regarding working units, nurses who were working in the emergency department scored the lowest in knowledge score compared to nurses in other departments. However, nurses who were working in the pediatric and gynecology departments showed the highest scores of knowledge (17.67) among other departments followed by operation nurses with a mean knowledge score of (17.2). It is worth mentioning that the number of participated nurses from operation and pediatrics/gynecology was the lowest.

**Table 1:** Comparing nurses' knowledge of standard precaution with respect to demographics.

Variables		Knowledge score		Kruskal-Wallis Test		Mann-Whitney *U* test
		Mean	(SD)	x^2^ test	p-value	
Hospital	P. Basma H	13.83	3.02	156.90	<0.001	P. Basma H Vs. KAUH (P < 0.001)
	KAUH	18.72	1.19			KAUK Vs. Mafraq H (P < 0.001)
	Mafraq H	14.44	2.29			
Gender	Male	16.33	2.96			0.990
	Female	16.23	3.27			
Education	Bachelor	16.08	3.19			0.002
	Master	18.12	1.99			
Professional Rank	RN	16.28	3.22	0.009	0.996	
	In charge/senior	16.23	3.18			
	Head nurse	16.67	2.06			
Working unit	Medical/Surgical	16.50	3.02	22.417	<0.001	Emergency Vs. Medical (p = 0.001) Emergency Vs ICU (P = 0.002) Emergency Vs Pediatric (P < 0.001) Emergency Vs Operation (P = 0.002)
	Emergency	14.88	3.17			
	ICU/CCU	16.56	3.20			
	Pediatric/Gynecology	17.68	2.53			
	Operation	17.24	2.79			

SD: Standard Deviation; x^2^: Chi-square value; p-value: statistically significant value.

### 3.5 Comparisons of compliance according to demographical variables

Several comparisons were made between demographical groups to identify variations in compliance scores to standard precautions. As shown in Table 2, nurses who were working in the KAUH scored higher in compliance level compared to other governmental hospitals (p < 0.001).

**Table 2:** Comparing nurses' compliance to standard precaution with respect to demographics.

Variables		Compliance score		Kruskal-Wallis Test		Mann-Whitney *U* test
		Mean	(SD)	×2 test	p-value	
Hospital	P. Basma H	48.33	12.53	34.447	<0.001	P. Basma H Vs. KAUH (P = 0.005)
	KAUH	53.25	12.74			KAUK Vs. Mafraq H (P < 0.001)
	Mafraq H	42.03	6.89			
Gender	Male	49.78	12.22			0.545
	Female	48.68	12.48			
Education	Bachelor	48.68	11.96			0.144
	Master	53.64	15.59			
Professional Rank	RN	50.16	12.17	2.162	0.339	
	In charge/senior	47.79	11.99			
	Head nurse	52.42	17.10			
Working unit	Medical/Surgical	48.06	11.63	30.554	<0.001	Medical Vs. Pediatric (p = 0.001) Medical Vs. Operation (p = 0.002) Emergency Vs. ICU (p = 0.004) Emergency Vs. Pediatric (p < 0.001) Emergency Vs. Operation (p < 0.001) ICU Vs. Pediatric (p = 0.022) ICU Vs. Operation (p = 0.002)
	Emergency	44.81	12.13			
	ICU/CCU	49.95	11.58			
	Pediatric/Gynecology	57.45	15.04			
	Operation	55.28	9.04			

SD: Standard Deviation; ×2: Chi-square value; p-value: statistically significant value.

However, no differences were found in compliance scores between both genders, levels of education, and professional ranks. Like the knowledge scores, nurses working at the emergency departments gained the lowest compliance level compared to nurses in the pediatric/gynecology departments who gained the highest compliance level compared to other working units followed by operation nurses (Table 2).

### 3.6 Factors influencing compliance to standard precaution

This section demonstrated the relationships between knowledge and compliance of standard precautions and aimed to determine the factors that may affect the compliance of standard precautions. The first step aimed to define the strength of these relationships using a correlation matrix. As shown in Table 3, there were several acceptable correlations (above 0.30 is preferable) between these variables. Focusing on factors that may influence the level of compliance to standard precautions, it is apparent that there was a positive correlation between compliance and the following three variables: knowledge; age; and experience. However, because the magnitude of the relationship between compliance and age was below 0.30, it was recommended to exclude it from the following regression model.

**Table 3:** Inter correlation matrix of variables affecting compliance Spearman's rho Correlation.

	Knowledge	Compliance	Age	Experience
Knowledge	1.00			
Compliance	.387^a^	1.00		
Age	.264^a^	.262^a^	1.00	
Experience	.455^a^	.341^a^	.938^a^	1.00

a Correlation is significant at 0.01 level (2-tailed).

### 3.7 Regression model

As shown in Table 4, total knowledge and professional experience were assessed as predictors to the compliance (Independent variable) based on the above correlation results. The regression model showed that more than 17% of the variance of compliance was explained by both knowledge and experience. Further, the beta value of the standardized coefficient for both knowledge and experience was 0.310 and 0.191, respectively. This result indicated that there was at least a positive influence of knowledge of standard precautions and clinical experience on attaining the level of compliance to standard precautions.

**Table 4:** Standard Multiple Regression model of factors influencing compliance to standard precaution.

Model	R	R Square	Adjusted R Square	R Square change	F Change	df	Sig
1	.424^a^	.180	.173	.180	28.787	263	<0.001

Dependent variable: Total compliance score.

a Predictors (independent variables): Total knowledge score, experience in years.

## 4 Discussion

The results of this study indicated a high level of knowledge among the participants. The result showed a mean of a total knowledge score of about 16.27/20 (81.35%). It was evident that most of the participants' who responded to the standard precautions knowledge questionnaire tend towards accepting all practices except the last two items which focused on safety precaution for patients with tuberculosis, Varicella, intestinal infection, and skin infection, which were recorded as rejection/uncertain responses by participants. Our study revealed that 95.1% of the participants knew and realized what standard precautions represent, 91.4% agreed that standard precautions protect both patients and health care workers, and 94% (N = 250) believed that standard precautions should be applied for confirmed-diagnoses patients or in the latent period of patients’ infection. However, this contradicts with many studies in the region that were highlighting the urgent need to implement professional programs to improve knowledge on standard precautions [16,17,[30], [31], [32], [33], [34]].

Many previous studies showed that the level of compliance with standard precautions guidelines is low among health care workers [18,35]. However, our study participants showed an intermediate level of compliance where all nurses scored a mean of 49.15/80 (61.4%) in their compliance with standard precautions. On the other hand, the level of knowledge was considered high in comparison with the previous studies. Therefore, the findings of the current study showed that the level of compliance might be influenced by the level of knowledge as has been the case in other studies with intermediate to high compliance with the standard precautions [6,36].

On the other hand, the results of this study showed that the highest level of compliance among nurses was when they were contacting the mucosa of patients, followed by contacting impaired skin (25.2%). Unlike those results, others found that participants wore gloves when mainly withdrawing blood and when performing venous puncture [6,37].

Regarding compliance with other Personal Protective Equipment's (goggles, masks, and gown), the results of this study showed that 24.8% of total nurses usually wear a face mask to protect nasal and oral mucosa in procedures that might induce spraying of blood and body fluids and 33.5% sometimes wear a face mask. This result was in agreement with other previous studies [38]. On the other hand, for wearing a protective suit (gown), 6.8% of the participants always wore them, 12.0% usually, while 43.6% rarely wore a protective gown. Unfortunately, those percentages were very low compared to some other studies [39] and this issue needs special care on orientations, workshops, and educational courses. However, some other studies reported similarly low levels of compliance to personal protective types of equipment [8,38,40] which may be related to the shortage of those types of equipment in the departments.

When comparing the level of knowledge and level of compliance regarding needle recapping, the percent of participants who knew that used needles should not be recapped was higher than the percentage of participants who never recapped the used needles and the results were (74.8%, 34.6%), respectively. This gap might be due to that only 52.6% of Prince Basma nurses had standard precaution training and only 44.4% of Al Mafraq hospital nurses had the training, while 84.0% of KAUH nurses were trained about standard precautions. Other reasons might be due to the lack of supervision or ignorance of the needle stick injuries risks and possibilities in the transmission of bloodborne pathogens. The results of this study indicated that the level of knowledge among Jordanian registered nurses was higher than the level of compliance; this variation might be caused by several factors, such as lack of PPEs, forgetfulness to follow standard precautions, and workloads. As well, this noncompliance to standard precautions might be caused by the absence of role models from colleagues or superiors, and the heavy workload that nurses have in the hospitals [31]. Other barriers such as emergency settings, and negative side effects of the protective equipment on the skin, may also negatively affect nurses’ compliance [41].

The in-depth analysis showed that there was a positive correlation between compliance with standard precautions and the following three variables: knowledge of standard precautions, age of participants, and level of experience.

Regarding the relationship between the level of participants’ knowledge and the level of their compliance with the standard precaution guidelines, the key finding was a positive correlation between them, as shown in Table 3, and the strength of the relationship was (r = + 0.387, p ≤ 0.01). This indicated that increasing the level of knowledge will lead to an increase in the level of compliance towards the standard precautions and can improve the nurses' clinical practice. This finding was expected and in agreement with other studies [8,17,32,33,40]. Therefore, health care facilities need to arrange training sessions for all nurses and other health care providers to improve their knowledge and to improve their level of compliance [42]. It is worth mentioning that other similar studies showed a positive correlation with nurses' age and level of experience [8,18,43].

## 5 Conclusions

In the Arabic world and particularly “Jordan”, there is an urgent need for more studies concerning the knowledge and compliance with standard precautions guidelines as one of the most important health issues for both health care workers and patients. The current study found that registered nurses in the north of Jordan hospitals may have a high total knowledge score, however, the total score of compliance to standard precautions was moderate with a significant variation between different hospitals. The current study showed that there was a positive correlation between the level of knowledge and the level of compliance to standard precautions, and the factors that affected this compliance were: knowledge, age, and clinical experience. Therefore, it is critical for every health care facility to arrange training sessions for all nurses and other health care providers to improve their knowledge and to improve the level of compliance.

## 6 Limitations

Just like any other cross-sectional study has potential limitations. First, external validity may be threatened because of the selection bias when using a convenient sample and not using a randomized sampling method. This cross-sectional study was limited to two governmental hospitals and one university-affiliated hospital in the north of Jordan, and the results of this study can't be generalized for all Jordanian registered nurses because other regions in Jordan were not included and the private and military sectors were not included too. Also, there was only one observer to collect data and it was difficult to measure the nurses' performance on all three shifts equally and compare between them. Finally, due to workload in governmental hospitals, some participants do not complete the questionnaire for the first time, while some of the participants forgot to complete the questionnaire and some of them had lost their questionnaire.

## Ethical approval

This work was approved by the IRB committee at King Abdullah University Hospital.

## Source of funding

Not Applicable.

## Author contribution

All listed authors contributed to the study design, data collection, data analysis, interpretation, and writing the paper.

## Research registration number

Name of the registry: Research Registry.

Unique Identifying number or registration ID: researchregistry6454.

Hyperlink to your specific registration (must be publicly accessible and will be checked): https://www.researchregistry.com/browse-the-registry#home/.

## Guarantor

Dr. Ibrahim Al-Faouri.

Dr. Nasr Alrabadi.

## Consent

A consent form was signed by all participants.

## Provenance and peer review

Not commissioned, externally peer-reviewed.

## Declaration of competing interest

The author(s) declared no conflicts of interest.
